# Synthetic co-culture in an interconnected two-compartment bioreactor system: violacein production with recombinant *E. coli* strains

**DOI:** 10.1007/s00449-024-03008-1

**Published:** 2024-04-16

**Authors:** Tobias Müller, Simon Schick, Jan-Simon Klemp, Georg A. Sprenger, Ralf Takors

**Affiliations:** 1 Institute of Biochemical Engineering, University of Stuttgart, Stuttgart, Germany; 2 Institute of Microbiology, University of Stuttgart, Stuttgart, Germany

**Keywords:** Synthetic modular co-culture, Two-compartment bioreactor, Temperature sensitive, Bioprocess engineering, Violacein

## Abstract

The concept of modular synthetic co-cultures holds considerable potential for biomanufacturing, primarily to reduce the metabolic burden of individual strains by sharing tasks among consortium members. However, current consortia often show unilateral relationships solely, without stabilizing feedback control mechanisms, and are grown in a shared cultivation setting. Such ‘one pot’ approaches hardly install optimum growth and production conditions for the individual partners. Hence, novel mutualistic, self-coordinating consortia are needed that are cultured under optimal growth and production conditions for each member. The heterologous production of the antibiotic violacein (VIO) in the mutually interacting *E. coli*–*E. coli* consortium serves as an example of this new principle. Interdependencies for growth control were implemented via auxotrophies for L-tryptophan and anthranilate (ANT) that were satisfied by the respective partner. Furthermore, VIO production was installed in the ANT auxotrophic strain. VIO production, however, requires low temperatures of 20–30 °C which conflicts with the optimum growth temperature of *E. coli* at 37 °C. Consequently, a two-compartment, two-temperature level setup was used, retaining the mutual interaction of the cells via the filter membrane-based exchange of medium. This configuration also provided the flexibility to perform individualized batch and fed-batch strategies for each co-culture member. We achieved maximum biomass-specific productivities of around 6 mg (g h)^−1^ at 25 °C which holds great promise for future applications.

**Supplementary Information:**

The online version contains supplementary material available at 10.1007/s00449-024-03008-1.

## Introduction

The natural secondary metabolite violacein (VIO) and its derivatives demonstrate striking pharmacological properties against Methicillin-resistant *Staphylococcus aureus* strains [1], malaria parasites [2, 3], and viral pathogens [4, 5] amongst others. Moreover, the versatile functions of VIO also include the potential to induce tumor cell death [6, 7] or enhance antibody production in Chinese hamster ovary cells [8], suggesting an even wider range of applications in healthcare and pharmaceutical industries. Additionally, the UV-absorbing and dyeing properties of the natural pigment in combination with its antibiotic properties may lead to potential applications in the fields of cosmetic and textile production [9, 10]. The use of violacein in pigment-based whole-cell biosensor approaches for the detection of heavy metals such as mercury [11] and lead [12] is another application that takes advantage of its color properties and even extends the potential usage to applications in environmental pollution monitoring.

The well-established production chassis *E. coli* has proven to be particularly suitable for the heterologous VIO production. Using advanced metabolic engineering strategies, several groups reported VIO synthesis at the gram-scale [13–16], reaching titers up to 4.5 g L^−1^ [17]. Through the combined efforts of metabolic- and integrated membrane engineering, the highest reported accumulations of the hydrophobic pigments were achieved, resulting in over 8 g L^−1^ of crude VIO and even over 11 g L^−1^ of pure deoxyviolacein [18]. A recently published study [19] adopted a different approach following the emerging concept of division of labor in modular co-cultures for VIO synthesis. Although the absolute output was relatively low (< 50 mg L^−1^), the study indicated beneficial properties of the synthetic *E.coli* consortium compared to a mono-culture control.

Despite the biological and genetic background of the mono- and co-cultures used so far, all strains were grown at temperatures that are clearly below the optimal range for *E. coli*. This is because the heterologously introduced key enzymes for VIO synthesis have lower temperature optima [20–22]. This discrepancy could theoretically be addressed by enzyme engineering strategies. However, since the thermostability properties of an enzyme are determined by complex, multi-layered influences [23], the coordinated modification of all five VIO pathway enzymes is likely to be a very challenging task. Therefore, other strategies must be found to address this inherent temperature conflict. While a mono-culture approach only permits uniform temperature settings, the modular biosynthetic pathway distributed among a synthetic co-culture can, in principle, allow process engineering strategies that support metabolic subunits with different physicochemical requirements. Therefore, a uniform co-culture cultivation method does not take full advantage of the versatile development possibilities of consortia-based production [24]. Implementing a spatially separated fermentation strategy using a specialized modular co-culture production system could provide a more efficient solution for VIO synthesis. Such an approach would exploit the biochemical advantage of the division of labor of a co-culture while simultaneously providing optimal temperature conditions for the native metabolic needs of precursor and co-metabolite production and the heterologous VIO enzymes, respectively. Although compartmentalization approaches have been developed to e.g. simultaneously provide the required oxygen profiles for cellulose-degrading and bioproduct-forming organisms in consolidated processes [25, 26], such approaches may not be transferable to other abiotic factors, such as temperature, or production-relevant reactor systems, such as stirred-tank bioreactors (STRs).

In contrast to the often-implemented relationship principle of commensalism, mutualistic co-cultures could serve as a predestined biological framework for co-culture-based biomanufacturing. Due to their intrinsic interdependencies, such mutualistic systems allow a balanced metabolic coordination, making their use as a stable and resource-efficient production platform conceivable in the future [27–29].

In the light of these consideration, this study aimed to investigate the temperature-sensitive production of VIO using a synthetic mutualistic *E. coli—E. coli* co-culture in an interconnected two-compartment, dual-temperature level bioreactor system. Based on the biological and technical system outlined in complementary research [29] the basic concept can be outlined as follows. A L-tryptophan (TRP) auxotrophic anthranilate (ANT) producing strain and an ANT auxotrophic strain, the latter expressing heterologous enzymes for the TRP-derived VIO production at lower temperatures were cultured in two distinct stirred-tank bioreactors (STRs), also referred to as compartments. Both compartments were equipped with in situ filtration devices: As a result, inter-compartmental metabolite exchange was possible while maintaining spatial separation of the strains. This allowed for the establishment of individualized temperature and process strategies for each co-culture member, to optimally support the metabolic sub-cassettes of the overall VIO production through a modular co-culture of interdependent strains. Quantitative analysis of relative and absolute metabolite dynamics from different process strategy approaches provided insight into the beneficial properties and potential for optimizing VIO production using synthetic co-cultures in spatially distinct bioreactor environments.

## Materials and methods

### Strains and plasmids

Strains and plasmids of this study are listed in Table 1. The strain ANT-5 is based on *E.coli* K-12 strain ANT-3 (LJ110 Δ*trpD* Δ*tnaA::FRT* Δ*trpR::FRT*-*Km*^*R*^-*FRT*) and TRP-5 is based on TRP-3 (LJ110 Δ*trpE* Δ*trpR::FRT* Δ*tnaA::FRT*), respectively, as described previously [29]. Therein, ANT-5 produces ANT and is auxotrophic for TRP. The strain was made kanamycin-sensitive by removal of the *KmR*-cassette. It carries an additional disruption of Δ*araBAD::FRT* and a deletion of *lacZ* (Δl*acZ*). Strain TRP-5, as TRP-3, is auxotrophic for TRP but can be supplemented as well with ANT to satisfy its need for TRP. Further modifications (Δ*araBAD*) and the chromosomal integration of Δ*fuc::Ptac-vioD* were implemented via a plasmid-based CRISPR/Cas9 method as described earlier [30]. The *vioD* gene is from *Chromobacterium violaceum* ATCC 12472 and was codon-optimized for *E. coli* K-12. Plasmid pVio_empty_-km is an isogenic control vector to pVioABCE-km [31] and was created by removal of the *vioABCE* genes.

**Table 1:** Overview of strains and plasmids

Name	Relevant genotype	Reference/Source
Strains		
LJ110	*E. coli* K-12 W3110 *fnr*^+^	[32]
ANT-5	LJ110 Δ*trpD* Δ*tnaA::FRT* Δ*trpR::FRT* Δ*lacZ* Δ*araBAD::FRT* pVio_empty_-km	Institute of Microbiology, University of Stuttgart
TRP-4	LJ110 Δ*trpE* Δ*trpR::FRT* Δ*tnaA::FRT* Δ*fuc::P*_*tac*_*-vioD* pVioABCE-km	Institute of Microbiology, University of Stuttgart
TRP-5	LJ110 Δ*trpE* Δ*trpR::FRT* Δ*tnaA::FRT* Δ*fuc::P*_*tac*_*-vioD* Δ*araBAD* pVioABCE-km	Institute of Microbiology, University of Stuttgart
Plasmids		
pVioABCE-km	*P*_*BAD*_*-vioABCE Km*^*R*^	[31]
pVio_empty_-km	*vio* genes deleted from *pVioABCE, Km*^*R*^	Institute of Microbiology, University of Stuttgart

### Pre-culture medium and procedure

The same minimal medium as described in [29] was used for pre-culture growth. This medium was adapted from [33] and contained per liter: 3 g KH_2_PO_4_, 12 g K_2_HPO_4_, 5 g (NH_4_)_2_SO_4_, 0.1 g NaCl, 1.710 g sodium citrate ∙ dihydrate, 0.1125 g FeSO_4_ ∙ 7 H_2_O, 0.015 g CaCl_2_ ∙ 2 H_2_O, 0.3 g MgSO_4_ ∙ 7 H_2_O and 0.0084 g thiamin ∙ HCl. Furthermore, a trace element solution [34] was added (0.4 ml L^−1^). This trace element solution contained per liter: 2.8 g MnSO_4_ ∙ H_2_O, 2.5 g AlCl_3_ ∙ 6 H_2_O, 1.825 g CoCl_2_ ∙ 6 H_2_O, 0.5 g ZnSO_4_ ∙ 7 H_2_O, 0.5 g Na_2_MoO_4_ ∙ 2 H_2_O, 0.25 g CuCl_2_ ∙ 2 H_2_O, 0.125 g H_3_BO_3_. As carbon- and energy source, 5 g L^−1^ glucose was supplied and 0.05 g L^−1^ of either TRP or ANT was added to overcome the respective genetic deletions of the strains. Since both co-culture strains used in this work contained a plasmid carrying kanamycin resistance, the cultures of both strains were supplemented with kanamycin (0.05 g L^−1^ final concentration). The medium pH was initially set to 7.2. Before the inoculation of the main cultures, the respective pre-culture suspension volume was washed and the cells were resuspended in main culture medium (specified in 2.3 and 2.4 plus subchapters). For further details, please see [29].

### Supplemented shaking flask cultivations

Strain TRP-4 was cultivated in 50 mL minimal medium in 500 mL shake flasks with baffles in triplicates at 20 °C, 25 °C, 30 °C and 37 °C. The composition of the medium was the same as that of the pre-culture medium, except for the following deviations. 0.1 g L^−1^ ANT was supplemented and 5 g L^−1^ L-arabinose was provided instead of D-glucose. Furthermore, 0.1 mM IPTG was added. All flasks were inoculated to a starting OD_600_ value of 0.2.

### Fed-batch co-culture cultivation in a two-compartment bioreactor setting with filtrate exchange

In principle, the same bioreactor system including filtration modules as described in [29] was used in this study. Essentially, the system consisted of two 1.5 L stainless steel stirred-tank bioreactors, each equipped with a Rushton turbine and four baffles. The strain ANT-5 was cultivated in bioreactor 1 (R1) and TRP-5 was cultivated in bioreactor 2 (R2). The microfiltration modules (D-Series FISP, Rapid Flow 0.2 μm membrane; Flownamics, USA) installed in both bioreactors allowed for the transfer of cell-free medium between the two compartments using peristaltic pumps. The culture medium used was the same for all approaches, only the auxotrophic components ANT and TRP as well as the D-glucose concentration varied. The further composition of the media corresponded to that of the pre-culture medium, except for the following differences. A tenfold higher concentration of trace elements was used, 170 µL L^−1^ antifoam J647 (Schill + Seilacher “Struktol” GmbH, Germany) was added and the pH was adjusted to 7. The starting liquid volume was 700 mL, the air supply rate was 300–500 ml min^−1^ over the processing time, and the stirring speed was between 400 and 500 min^−1^. The dissolved oxygen content was kept above 30% by automated stirrer control and the system was operated without overpressure. The pH was kept at 7 by controlled addition of 25% (w/w) ammonia solution and 25% (w/w) H_3_PO_4_.

#### Process strategy I

For Process Strategy I, both bioreactors were inoculated at the same time to an OD_600_ starting value of 0.1. The medium for R1 contained 3.5 g L^−1^ D-glucose and 0.035 g L^−1^ TRP, the medium for R2 also contained 3.5 g L^−1^ D-glucose but 0.022 g L^−1^ ANT. The supplemented batch phase was run at 37 °C in both bioreactors. After 8.8 h process time, the filtrate exchange (0.6 mL min^−1^) was initiated between the two bioreactors by switching on the connecting peristaltic pumps. Also, the temperature in R2 was lowered to 25 °C and the inducers L-arabinose and IPTG were added to both bioreactors to set a media concentration of 5 g L^−1^ and 0.1 mM, respectively. Simultaneously, the feed media supply for both bioreactors was started (R1: µ_set_ = 0.1 h^−1^; D-glucose 150 g L^−1^, L-arabinose 5 g L^−1^, IPTG 0.1 mM; R2: µ_set_ = 0.05 h^−1^; D-glucose 50 g L^−1^, L-arabinose 5 g L^−1^, IPTG 0.1 mM).

#### Process strategies II and III

The medium in R1 contained a starting D-glucose concentration of 7 g L^−1^ and 0.07 g L^−1^ TRP and was inoculated to a starting OD_600_ of 0.3. R1 was operated entirely at 37 °C. After 7.2 h process time, a constant feed supply was started (0.05 ml min^−1^; D-glucose 187 g L^−1^, L-arabinose 5 g L^−1^, IPTG 0.1 mM).

R2 was inoculated 4.9 h after R1 to a starting OD_600_ of 0.03. In R2, the initial D-glucose concentration in the medium was 3.5 g L^−1^, and ANT was introduced at a concentration of 0.022 g L^−1^. The supplemented batch phase was also performed at 37 °C in R2. After 13.9 h of process time for R2 (corresponding to 18.8 h of process time for R1), the filtrate exchange between the two bioreactors (0.6 ml min^−1^) was started by switching on the connecting peristaltic pumps. Additionally, the temperature in R2 was reduced to 25 °C. At the same time, both bioreactors were also provided with an inductor pulse to reach a concentration of 5 g L^−1^ L-arabinose and 0.1 mM IPTG. The constant feeding rate in R1 was reduced to 0.02 ml min^−1^, and the feed supply in R2 was initiated (µ_set_ = 0.05 h^−1^; D-glucose 80 g L^−1^, L-arabinose 5 g L^−1^, IPTG 0.1 mM). After 18.1 h filtrate exchange time, the filtrate flux between the bioreactors was reduced to 0.3 ml min^−1^. Process Strategy III was identical to Process Strategy II except that the temperature in R2 was lowered to 30 °C instead of 25 °C.

### Analytical methods

The cell dry weight (CDW) concentration was determined by photometric measurement of the optical density at a wavelength of 600 nm (OD_600_) and using the correlation factor CDW [g L^– 1^] = OD_600_ • 0.28, as described in previous work [29]. In addition, the method used to quantify D-glucose concentrations was performed according to the information provided in the said publication, the method to quantify ANT and TRP was slightly modified (see Supplementary Information). Adapted from [13], the sampled cell pellets were repeatedly extracted with pure ethanol under glass bead cell disruption (6000 rpm, 30 s; Precellys24, Bertin Technologies SAS, France; d_Glass beads_ = 0.5 mm; Carl Roth GmbH + Co. KG) for the quantification of VIO. The extraction phases were pooled, and the remaining solids were centrifuged off (5 min, 20,817 g, 4 °C). Violacein and deoxyviolacein were quantified using a HPLC method as described by [35]. For this purpose, a UHPLC (UltiMate 3000-Series; Dionex; Thermo Fisher Scientific Ind., USA) with a C-18 column (Luna C18(2), 5 µm, 250 × 4.6 mm; Phenomenex Inc, USA) at 30 °C was used. 50%(v/v) ethanol at a flow rate of 0.5 ml min^−1^ was used as mobile phase. The autosampler was cooled (5 °C), and the injection volume was 10 µL. A diode array detector (DAD-3000) was used for signal detection at 258 nm and 580 nm, corresponding to values at or near the absorption maxima of the pigments in ethanol [36]. Quantification was based on external standards dissolved in pure ethanol, prepared from commercially purchased violacein (Cayman Chemical Company, USA) and deoxyviolacein (Santa Cruz Biotechnology, Inc., USA). VIO values given in this work are to be interpreted as crude violacein values and indicate the sum of violacein and deoxyviolacein.

### Filtrate flux estimation and verification of cell separation

Filtrate transfer between the bioreactors was determined gravimetrically. For this purpose, filtrate flux was completely branched off into preweighed tubes for 5 min. The filtrate flux was calculated from the weight difference and an assumed density of 1 g mL^−1^. With the start of filtrate exchange, cell suspensions from both bioreactors were collected at all sampling time points, diluted with NaCl (0.9% (w/v)), and plated on MacConkey agar (10 g L^−1^ lactose). Based on the deletion of *lacZ* in ANT-5, the two co-culture strains could be distinguished on the selection medium. ANT-5 colonies appeared pale, TRP-5 colonies showed a red staining.

### Calculation of rates and ratios

The biomass-specific VIO formation rates ${{\text{q}}}_{{\text{VIO}}}$ [mg (g h)^−1^] were determined differentially between two time points. For the determination of the synthesis rates under supplemented batch conditions in the shake flask, the change in VIO concentration $\Delta {{\text{c}}}_{{\text{VIO}}}$[mg L^−1^] was related to the mean biomass concentration ${\overline{{\text{c}}} }_{{\text{X}}({\text{TRP-4}})}$ [g L^−1^] and the corresponding time difference of the points considered $\mathrm{\Delta t}$ [h] (Eq. 1).

1 $${{\text{q}}}_{{\text{VIO}}}=\frac{\Delta {{\text{c}}}_{{\text{VIO}}}}{{\overline{{\text{c}}} }_{{\text{X}}({\text{TRP-4}})}\cdot \mathrm{\Delta t}}$$

As shown in Eq. (2), the biomass-specific VIO production rate calculation for TRP-5 ${\overline{{\text{c}}} }_{{\text{X}}({\text{TRP-5}})}$[g L^−1^] in the two-compartment production bioreactor additionally included the volume effect of the mean cell-free filtrate exchange flows between the bioreactors ( ${\overline{{\text{Q}}} }_{{\text{R1}}\to {\text{R2}}}$, ${\overline{{\text{Q}}} }_{{\text{R2}}\to {\text{R1}}}$; [L h^−1^]) as well as the mean D-glucose-containing feed for R2 ${\overline{{\text{F}}} }_{{\text{R2}}}$[L h ^−1^] on the mean VIO concentration ${\overline{{\text{c}}} }_{{\text{VIO}}}$ [mg L^−1^] in the production bioreactor.

2 $${{\text{q}}}_{{\text{VIO}}}=\frac{\frac{\Delta {{\text{c}}}_{{\text{VIO}}}}{\mathrm{\Delta t}} + \frac{\left({\overline{{\text{Q}}} }_{{\text{R1}}\to {\text{R2}}} + {\overline{\text{F}} }_{\text{R2}} - {\overline{{\text{Q}}} }_{{\text{R2}}\to {\text{R1}}}\right)}{{\overline{{\text{V}}} }_{{\text{R2}}}} \cdot {\overline{\text{c}} }_{\text{VIO}}}{{\overline{{\text{c}}} }_{{\text{X}}({\text{TRP-5}})}}$$

For the analysis of ANT dynamics, we studied the fraction of ANT that was consumed from the total amount of being supplied to the cells. The latter was termed ${{\text{m}}}_{{\text{ANT}},{\text{Present}},\text{R2}}$ [mg] and was calculated for each time interval as the amount of ANT transferred via filtration (using mean ANT concentrations (${\overline{{\text{c}}} }_{{\text{ANT}},{\text{R1}}}$, ${\overline{{\text{c}}} }_{{\text{ANT}},{\text{R2}}}$; [mg L^−1^] and filtrate flux) plus the mean ANT mass ${\overline{{\text{m}}} }_{{\text{ANT}},{\text{R2}}}$ [mg] which was already in R2 (Eq. 3).

3 $${{\text{m}}}_{{\text{ANT}},{\text{Present}},\text{R2}}=\left({\overline{{\text{Q}}} }_{{\text{R1}}\to {\text{R2}}}\cdot \mathrm{\Delta t}\cdot {\overline{\text{c}} }_{\text{ANT,R1}}\right) - \left({\overline{{\text{Q}}} }_{{\text{R2}}\to {\text{R1}}}\cdot \mathrm{\Delta t}\cdot {\overline{\text{c}} }_{\text{ANT,R2}}\right) + {\overline{\text{m}} }_{\text{ANT,R2}}$$

The ANT uptake is called ‘mass uptake capacity’ ${{\text{m}}}_{{\text{ANT,Uptake capacity,TRP-5}}}$ [mg] indicating that we took the maximum, estimated ANT uptake rate per time interval for calculations. As shown in Eq. (4), this value was calculated by multiplying a set biomass-specific ANT uptake rate ${{\text{q}}}_{{\text{ANT}}}$  [mg (g h)^−1^] with the mean biomass ${\overline{{\text{m}}} }_{{\text{X}}({\text{TRP-5}})}$ [g] and the respective time interval. Due to the diffusion-dependent characteristics of cellular ANT uptake, ${{\text{q}}}_{{\text{ANT}}}$ was determined in the time interval around the maximum ANT peak in R2 (between 12 and 17.1 h after induction) for process strategies II and III according to Eq. (5). Since a significant ANT accumulation is not detectable for Process Strategy I, the mean ${{\text{q}}}_{{\text{ANT}}}$ value determined for Process Strategy II was set.

4 $${{\text{m}}}_{{\text{ANT,Uptake capacity,TRP-5}}}={{\text{q}}}_{{\text{ANT}}}\cdot {\overline{\text{m}} }_{\text{X(TRP-5)}}\cdot \mathrm{\Delta t}$$

5 $${{\text{q}}}_{{\text{ANT}}}=\frac{-\frac{\Delta {{\text{c}}}_{{\text{ANT}}}}{\mathrm{\Delta t}}+\frac{{\overline{{\text{Q}}} }_{{\text{R1}}\to {\text{R2}}}}{{\overline{{\text{V}}} }_{{\text{R2}}}}\cdot \left({\overline{\text{c}} }_{\text{ANT,R1}}-{\overline{\text{c}} }_{\text{ANT,R2}}\right)-\frac{{\overline{\text{F}} }_{\text{R2}}}{{\overline{{\text{V}}} }_{{\text{R2}}}} \cdot {\overline{\text{c}} }_{\text{ANT,R2}} }{{\overline{{\text{c}}} }_{{\text{X}}({\text{TRP-5}})}}$$

Accordingly, the ratio ${{\text{f}}}_{{\text{ANT}},\mathrm{ Ratio},\text{R2}}$ [ −] that describes the degree of ANT saturation was calculated as shown in Eq. (6):

6 $${{\text{f}}}_{{\text{ANT}},\mathrm{ Ratio},\mathrm{ R2}}=\frac{{{\text{m}}}_{{\text{ANT}},\mathrm{ Presented}}}{{{\text{m}}}_{{\text{ANT}},\text{ Uptake capacity}}}$$

## Results and discussion

### Finding the temperature optimum for VIO production

Different psychrophilic and mesophilic native VIO producers show broadly varying temperature optima for pigment synthesis [37]. However, the heterologous synthesis of VIO in producer hosts requires a holistic temperature consideration that includes native precursor and co-substrate synthesis in addition to the heterologous VIO enzymes. Reported high-performing heterologous VIO-producing mono-cultures transferred *Duganella* sp. B2 (now classified as *Massilia violaceinigra* [38])-based *vio* genes into an *E. coli* production concept at 20 °C [14, 15, 17]. Using the same production chassis but inserting key *vio* genes derived from *C. violaceum*, one study identified a temperature optimum at 20 °C [31], while another group chose a production temperature of 30 °C [19, 39].

Therefore, in order to determine the temperature dependence of the VIO synthesis for the VIO-producing strain of the co-culture studied in this work, different production temperatures were evaluated. Experiments were performed with L-arabinose as the carbon source to ensure that the catabolite repression of the D-glucose-based production on the L-arabinose-induced promoter would not influence the validity of the temperature variation (as done before in [31]). Accordingly, a version of the producer strain without *araBAD* deletion was used (TRP-4). The extra supply with ANT simulated cross-feeding via the co-culture, enabling the investigation of growth and production of the auxotrophic VIO producer in mono-culture.

**Fig. 1:**
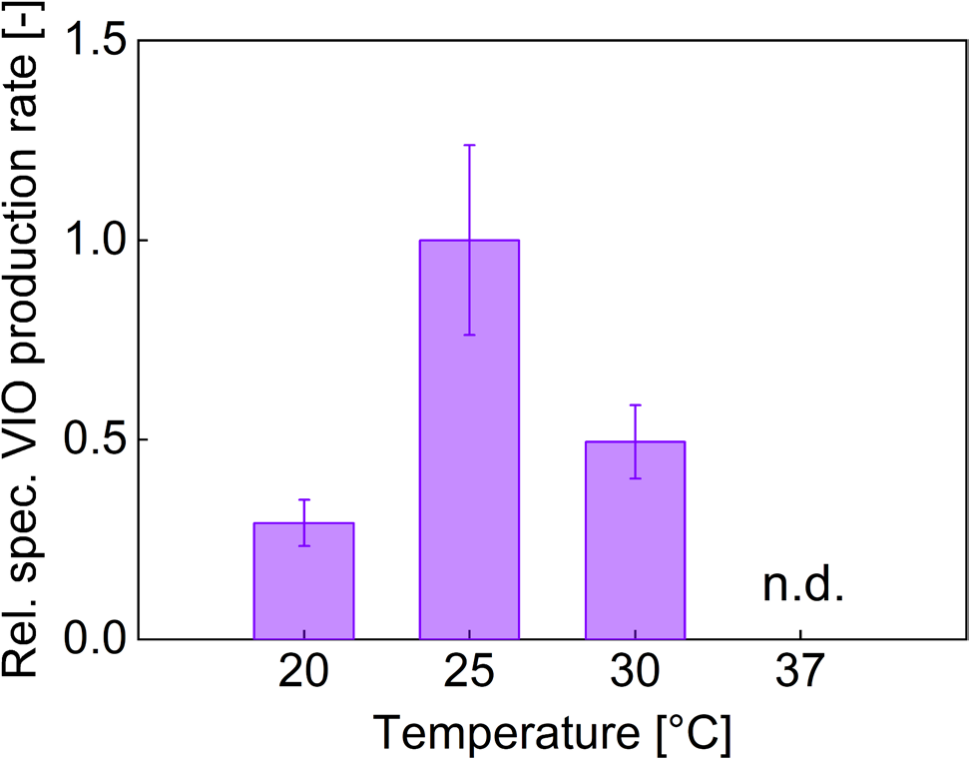
Biomass-specific crude violacein (VIO) production rates of strain TRP-4 in shaking flask cultivations (5 g L^−1^ arabinose and 0.1 g L^−1^ anthranilate) under different temperature settings, relative to the highest mean value (25 °C approach). The production rates were calculated between 0 and 96 h of cultivation time for the 20 °C setting, and between 0 and 27 h for 25 °C and 30 °C cultivations. At 37 °C, no violacein was detected at any time point. (See supplementary Information, Fig. S1). Abbreviation: *n.d.* Not detectable. Error bars indicate the standard deviation of biological triplicates

Considering growth dynamics and VIO production performance under the tested conditions, a temperature range of 25–30 °C turned out to be advantageous over 20 °C (Supplementary Information, Fig. S1). The highest biomass-specific VIO production rate was found at 25 °C, which on average was twice as high as the rate at 30 °C and 3.4 times higher than the specific production rate at 20 °C (Fig. 1). VIO formation could not be detected at 37 °C.

### Evaluating fed-batch modes

**Fig. 2:**
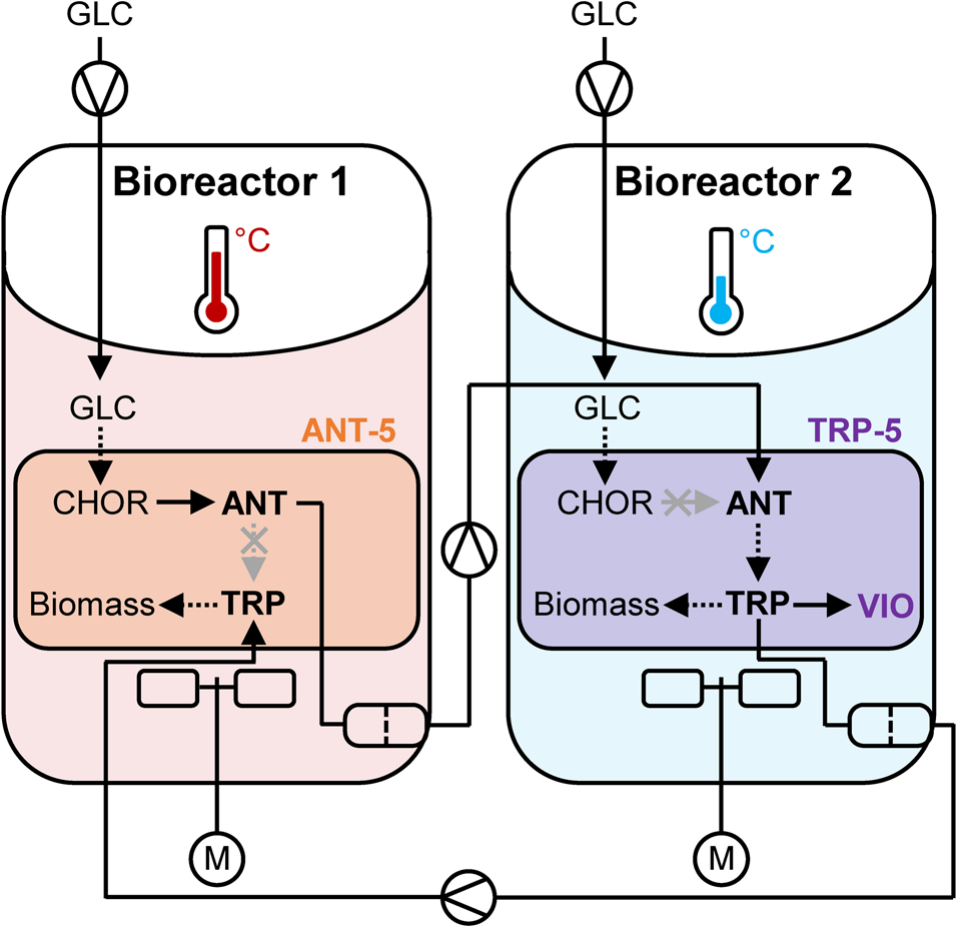
Schematic representation of the two-compartment bioreactor system and the spatially separated strains of the synthetic crude violacein (VIO)-producing co-culture. Only selected essential aspects of the bioreactor setups and the synthetic mutualistic co-culture organisms ANT-5 and TRP-5 are shown in simplified form. For the bioreactors, the corresponding temperature regimes are color-coded, the filter-mediated filtrate exchange and the fed-batch feedings are indicated. For the strains, only the key deletions (crosses) affecting the L-tryptophan (TRP) and anthranilate (ANT) auxotrophies are shown, the auxotrophic dependencies are clarified, and the heterologous VIO production capacity is depicted. Within the metabolic schemes, solid arrows represent direct conversions and dashed arrows represent lumped reactions. All detailed information regarding bioreactor and process specifications as well as strain manipulations can be found in the corresponding material and methods sections. Other abbreviations: *GLC* D-glucose, *CHOR* Chorismate, *M* Stirrer motor

The biological core of the VIO production system is a synthetically designed *E. coli* co-culture with an engineered mutual dependency in continuation of our earlier report [29]. The microbial consortium consisted of a TRP auxotrophic ANT-producing strain which was combined with an ANT-auxotrophic TRP-producing partner. This mutualistic consortium framework, based on division-of-labor through bi-directional metabolite exchange, has already demonstrated its applicability in the two-compartment bioreactor setting with bi-temperature level cultivation [29]. Therefore, heterologous VIO genes were additionally introduced into the TRP-producing strain to fully exploit the VIO production potential of the mutualistic approach (See Table 1). The spatial separation enabled the parallel installation of the identified optimum reduced VIO production temperatures in the production bioreactor and the equally optimum growth temperature for the ANT producer of 37 °C in the precursor bioreactor. Furthermore, independent batch and fed-batch conditions could be installed and the timing of the on-demand cell-free medium exchange between the two compartments was another adjustment parameter. An overview of the biological- and technical setting is depicted in Fig. 2.

**Fig. 3:**
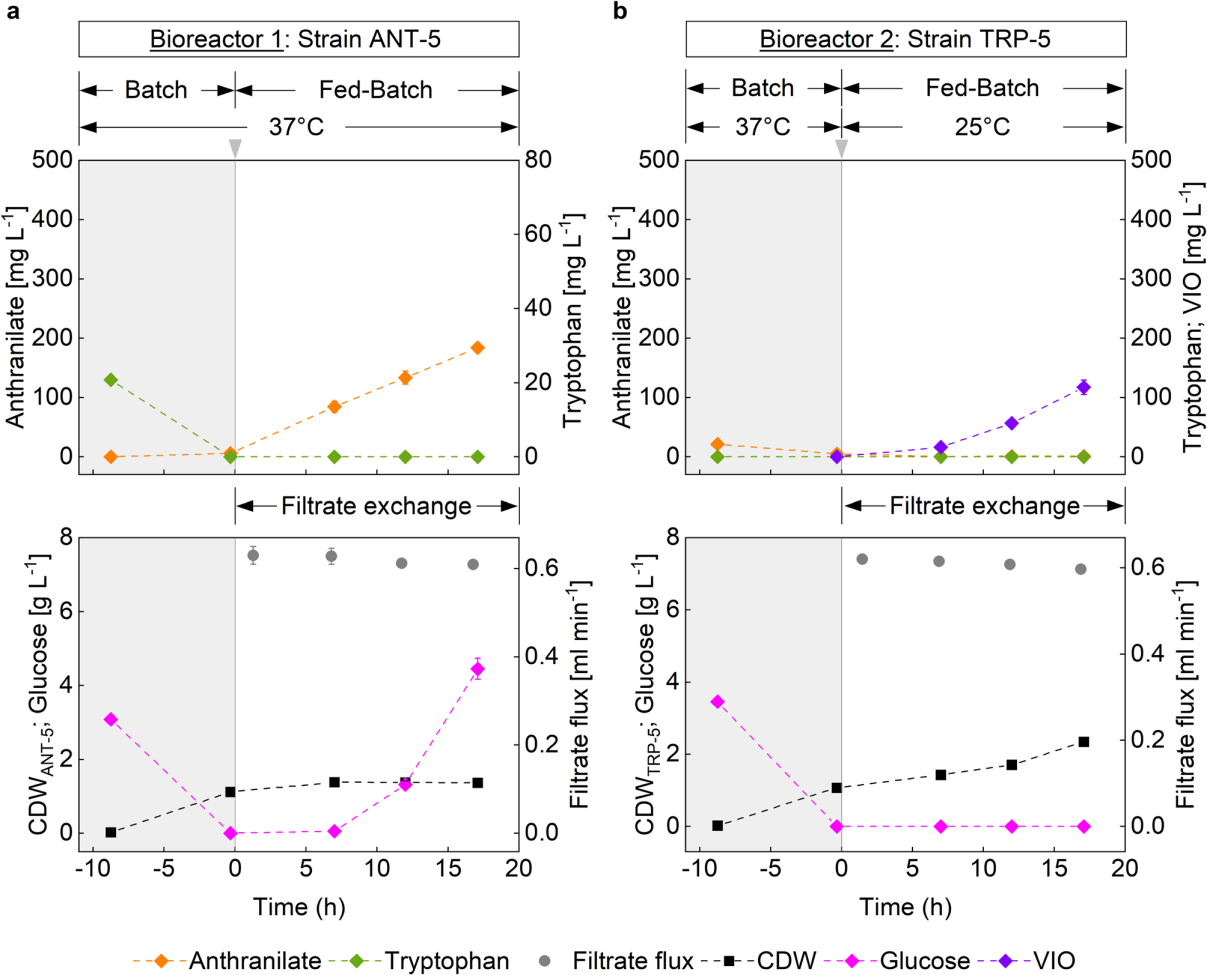
Process Strategy I—Time courses of biomass (CDW)- and extracellular metabolite concentrations in fed-batch production of crude violacein (VIO) by a spatially separated co-culture in an interconnected two-compartment bioreactor system. (**a**) Representation of the concentration curves, as well as the filtrate flux from bioreactor 1 (Harboring strain ANT-5). (**b**) Representation of the concentration curves, as well as the filtrate flux from bioreactor 2 (Harboring strain TRP-5). The gray areas correspond to the supplemented cultivation phase before filtrate exchange, inductor addition (grey arrows) and temperature change in bioreactor 2. For process details, see *Process Strategy I* in the *Materials and methods* section. Error bars indicate the standard deviation of biological duplicates

Several process designs were investigated to optimize VIO synthesis in this experimental setting. The first considered the parallel growth of both cultures at 37 °C. Related auxotrophic components were initially supplemented to reach equilibrated biomass concentrations (Fig. 3, gray areas). Subsequently, the filtrate exchange-based process phase was started (Fig. 3, blank areas). The temperature in R2, containing the VIO producer, was reduced to 25 °C, while the temperature in R1, containing the ANT producer, remained at 37 °C. In addition, inducer substances for *vio* gene expression were added. Both bioreactors were fed with a D-glucose-containing feed medium using exponential profiles. With the consumption of the supplied TRP and the start of feeding in R1, ANT-5 began to overproduce ANT. Filtrate exchange between the bioreactors allowed TRP-5 to synthesize ANT-based TRP in R2, which was channeled into biomass and VIO synthesis. The supplied ANT was sufficient to achieve the growth rate set by the glucose-feed (µ_TRP-5_ = 0.05 ± 0.00 h^−1^; 0 h-17.1 h). Only marginal residual amounts of ANT were found in the medium of R2, and no excess TRP was detected. We concluded, that the TRP auxotrophic strain ANT-5 in R1 was not sufficiently supplied with TRP, thus finally limiting growth. Remarkably, however, the resting ANT-5 cells used the supplied carbon source to synthesize a substantial amount of ANT, while excess D-glucose accumulated to a large degree in the medium.

**Fig. 4:**
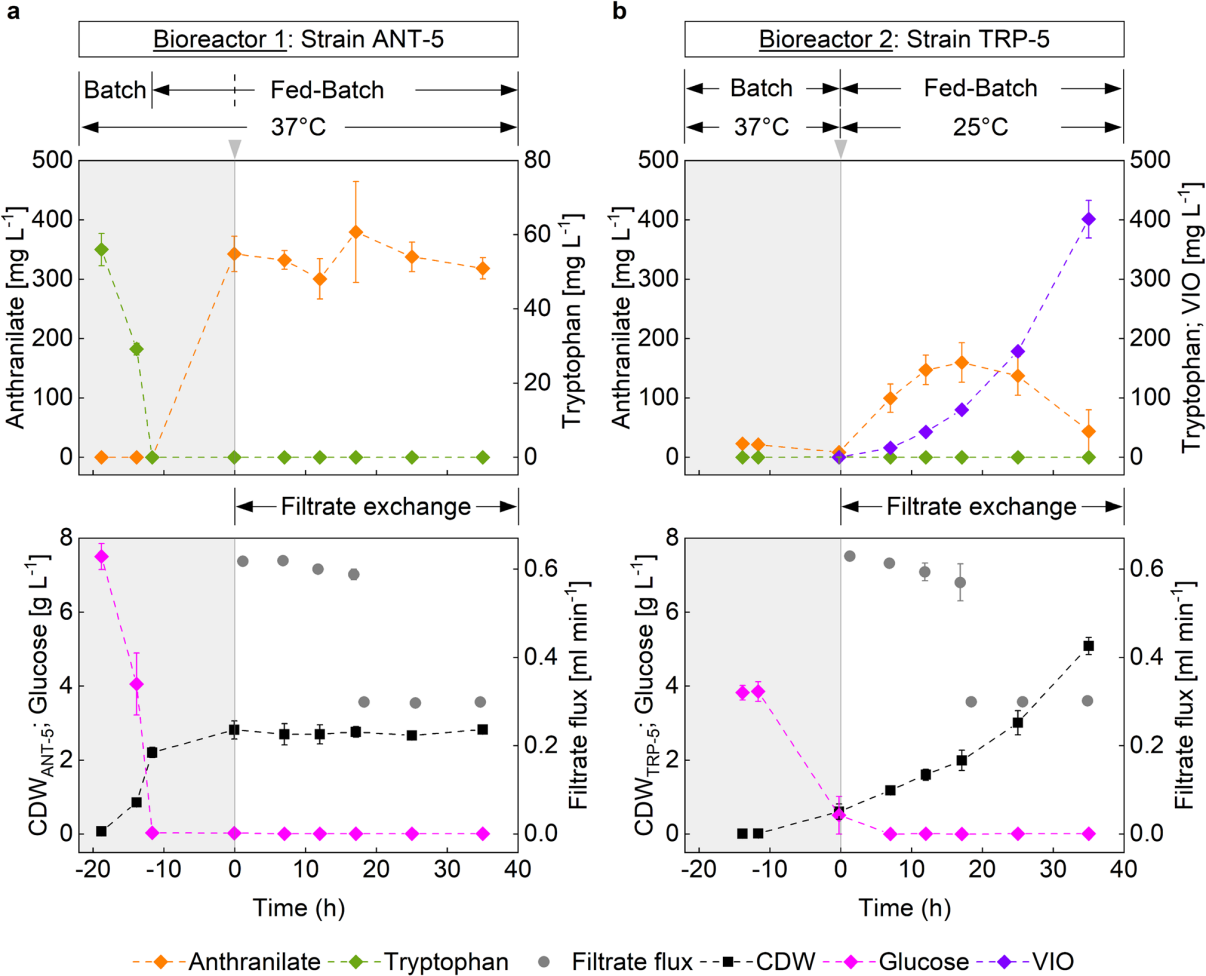
Process Strategy II—Time courses of biomass (CDW)- and extracellular metabolite concentrations in fed-batch production of crude violacein (VIO) by a spatially separated co-culture in an interconnected two-compartment bioreactor system. (**a**) Representation of the concentration curves, as well as the filtrate flux from bioreactor 1 (Harboring strain ANT-5). The dashed line within the fed-batch phase indicates the time point of the D-glucose feed-rate adjustment. (**b**) Representation of the concentration curves, as well as the filtrate flux from bioreactor 2 (Harboring strain TRP-5). The gray areas correspond to the supplemented cultivation phase before filtrate exchange, inductor addition (grey arrows) and temperature change in bioreactor 2. For process details, see *Process Strategies II and III* in the *Materials and methods* section. Error bars indicate the standard deviation of biological duplicates

The results of the first experimental setting motivated us to increase the ANT supply for the VIO producer. We assumed that ANT uptake is concentration dependent, which demands an increase of the ANT levels in R2 for boosting TRP and subsequent VIO production. Therefore, several process adjustments were made leading to Process Strategy II (see Fig. 4): Compared to Process Strategy I, the biomass of the ANT-5 producer was doubled through adjusted batch conditions. Additionally, the original exponential D-glucose feed was replaced by a two-level constant fed-batch profile to improve matching with the real microbial need. Furthermore, the initiation of filtrate exchange was shifted.

As a consequence, ANT strongly accumulated in R1, as expected, before the filtrate exchange started. After this onset, an almost stable ANT level could be achieved for the rest of the fermentation preventing D-glucose wastage at the same time. However, as indicated in Fig. 4, the supply of ANT exceeded the maximum ANT uptake of TRP-5 finally causing ANT accumulation in R2. During the first 17.1 h of the filtrate exchange-based process, the actual filtrate flux-rates between the bioreactors progressively decreased from the set value, likely due to biomass deposits on the filters. To prevent further blockage of the filters, the exchange fluxes were reduced after 18.1 h. Therefore, the fraction of consumed ANT in R2 increased leading to decreasing ANT levels in the medium of R2. The VIO producer TRP-5 was able to synthesize ANT-based VIO with a final titer of 401 ± 32 mg L^−1^.

Based on the preselected temperature range of 25 °C to 30 °C (see shaking flask results), Process Strategy II was further replicated using a production temperature of 30 °C instead of 25 °C (Process Strategy III; see Supplementary Information Fig. S2). Under the application relevant glucose-based fed-batch conditions, the elevated temperature resulted in reduced VIO accumulation, which was consistent with the arabinose-based shaking flask performances.

Finally, it should be noted that the integrity of the filter systems was always found to be intact, i.e. cross-contaminations of strains were prevented. This was experimentally proven by plating on Mac-Conkey lactose plates, which confirmed the sole existence of pure cultures in both bioreactors throughout the whole process.

### Process elucidation by analysis of rates

**Fig. 5:**
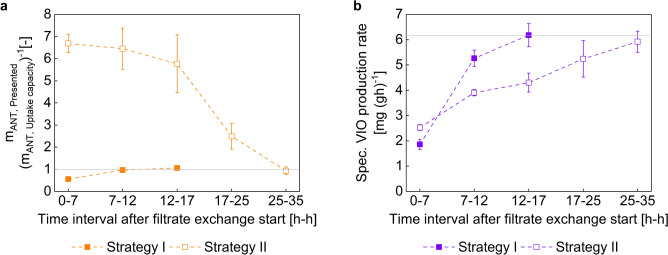
Quantitative consideration of relative anthranilate (ANT) dynamics and absolute crude violacein (VIO) production rates of TRP-5 in bioreactor 2 after the initiation of the induced filtrate exchange period. (**a**) Calculated mass ratio of the presented ANT and the uptake capacity within specific time periods (≙ f_ANT, Ratio, R2_). The horizontal reference line marks the balanced state of both elements. (**b**) Biomass-specific VIO production rates within specific time intervals. The horizontal reference line corresponds to the highest identified value. The filled squares represent the ratios and rates of Process Strategy I (See Fig. 3). The blank squares represent the ratios and rates of Process Strategy II (See Fig. 4). Time intervals are given in full hours, the difference in the total time span considered is due to the different process durations (See Figs. 3 and 4). Details on calculation can be found in the *Materials and methods* section. Error bars indicate the standard deviation of biological duplicates

For investigating the connection of filtrate-exchanged precursor supply, and product formation, related plots are given in Fig. 5. Figure 5a depicts the ratio ${{\text{f}}}_{{\text{ANT}},\mathrm{ Ratio},\mathrm{ R}2}$ [ −] which indicates the degree of ANT saturation for the strain TRP-5. Hence, the values may be interpreted as the x-fold saturation of ANT needs for VIO production in R2. For completion, Fig. 5b shows the biomass-specific VIO production rates for the same production scenarios.

The analysis of the ANT supply: uptake ratio, as shown in Fig. 5a, interprets the calculated values according to three scenarios. Values < 1 indicate a situation where the supply of ANT precursors was less than the amount that could be assimilated and subsequently metabolized. A value = 1 represents a balance between supply and absorption capacity, while values > 1 imply to an overprovision of ANT. It is important to note that ANT uptake was assumed to be diffusion-limited. Consequently, values not only < 1, but also potentially ≈ 1, may already indicate limitations.

After an initial period of undersupply of ANT, the supply situation in Process Strategy I improved towards a more balanced state. By the end of the processes, the ratio of Process Strategy II also reached a balanced state. In contrast, the ANT mass ratios for Process Strategy II was >  > 1 for the other periods.

When comparing both strategies it became obvious that the ANT oversupply in Process Strategy II, compared to the shortage in Process Strategy I, enabled initially increased specific VIO productivity. However, the continuous oversupply of ANT in the later course of Process Strategy II did not yield any benefits for the specific VIO productivity, even the opposite was the case. In a situation where the ANT ratio was balanced, Process Strategy I and Process Strategy II showed a similar maximum specific VIO production rate of 6.2 ± 0.5 mg (g h)^−1^ and 5.9 ± 0.4 mg (g h)^−1^, respectively.

A possible explanation for this phenomenon could be that increased ANT presentation may be unfavorable to TRP-5 leading to higher intracellular TRP pools. The *trpL* attenuator still being present in TRP-5 could thus have had a TRP-dependent feedback inhibitory effect on TRP/VIO synthesis. Furthermore, it seems plausible to consider the observed correlations in the light of the proposed hypothesis of an unknown, TRP-dependent pathway regulation [40].

Although the precursor availabilities of Process Strategies II and III were similar, the specific VIO productivities at 25 °C were consistently higher than those at 30° (see Supplementary Information, Fig. S3). This result was consistent with the preliminary shaking flask VIO module temperature sensitivity evaluations.

In summary, it has been confirmed that in addition to providing a favorable temperature-reduced environment of 25 °C, ANT should be available in sufficient, but not excessive, amounts at all times. The rationale for this was that maximum specific VIO productivity depends on the delicate interplay between ANT pool, exchange, and uptake capacity. A state of equilibrium between the available and the uptake mass per time appeared to allow for enhanced VIO synthesis rates. Therefore, to fully exploit the potential of the dormant precursor producer demonstrated in this study, the focus could shift to demand-driven ANT supply strategies to the production strain. By installing a filtrate exchange profile, dependent on VIO producer biomass dynamics, rather than a constant flow, a superior process performance could be achieved. A more elegant approach would be a filtrate exchange concept, directly coupled to ANT level in R2. A potential implementation strategy could be an ANT-inducible gene expression system coupled with e.g. fluorescence markers (similar like shown by [41]).

Interestingly, this type of synchronized metabolite exchange dynamics, which was identified as beneficial for VIO synthesis, served as one of the rationales for the development of our mutualistic co-culture concept. Thus, it is conceivable that the pathway leading to TRP and the subsequent pathway leading to VIO should be further manipulated to promote the re-feeding of the auxotrophic compound to the partner organism. With a considerable increase in filtrate exchange rates, it might be feasible to apply fitting process conditions, re-focusing on the inherently interconnected characteristics and making use of the resulting well-balanced metabolite exchange patterns for improved production processes.

The use of specially engineered *E. coli*–*E. coli* co-cultures have proved to be a valuable tool for elucidating the VIO synthesis pathway in detail [42]. In a recent study, Gwon et al. [19] showed that the application of a synthetic co-culture system of two *E. coli* strains can achieve superior VIO production compared to a reference mono-culture. The authors constructed a co-culture system in which both partner strains were able to synthesize TRP independently on their own. One strain additionally harbored heterologous VIO genes, allowing the strain to metabolize internal and partner-supplied TRP to VIO, which was shown in 96-well plate and shaking flask cultures. With this linear (“one-way”) relation of the biosynthesis modules and the cultivation of both strains in a shared environment (“one-pot”), this co-culture design follows the same concept as the majority of microbial biomanufacturing consortia reported so far [43].

In contrast, our research aims to harmonize not only the dynamics of co-culture composition and metabolite exchange but also the implementation of the overarching biological concept with the unique process requirements of the partner strains. In particular, we wanted to extend the concept explored in our previous study [29] to the temperature sensitive VIO synthesis as an example. Thus, our approach is based on a biological system that exhibits a true division of labor and synthetically induced, interdependent metabolite exchange behavior through complementary auxotrophies. We have introduced a degree of flexibility into this biological concept through spatial compartmentalization of the partner strains in two distinct STRs, connected by in-line filter modules to account for metabolite exchange. Similar to dialysis membrane reactors [44, 45], this strategy enabled straightforward strain-specific characterizations. Beyond that, the two-bioreactor system allowed for the installation of individual temperature and process adaptations which are unattainable in classic setups with a common environment. Our integrative co-culture approach resulted in an average specific VIO production rate which can be estimated to be approximately twice as high compared to the VIO producer in the “one-way”, “one-pot” co-culture approach by [19]. The achieved maximum specific VIO production rate under optimal process conditions highlighted an even greater potential. The two-compartment, dual-temperature co-culture approach represents a promising basis not only compared to other co-culture concepts, but also to the highly-modified mono-culture producers reported. The comparison to the production processes reported in [13, 16, 31] revealed that although our approach achieved a titer up to 4 times lower, the biomass-specific production rates can be estimated to be higher. However, other monoculture approaches have shown even substantially higher titers, along with apparently considerably higher rates [14, 15, 17, 18]. It is important to note that the studies compared here did not explicitly provide biomass-specific VIO production rates. Therefore, an approximate estimation based on data extracted from the studies was calculated for comparison purposes (similar to Eq. (1), assuming CDW-OD_600_ correlation factor as defined in 2.5 if necessary).

To exceed these benchmark values, the biological and technical co-culture framework presented in this study, needs further engineering. This can be achieved by targeting the already elaborated precursor balancing strategies and complementing them with further metabolic- and membrane engineering efforts to enhance TRP and VIO synthesis. Additionally, an in situ product removal strategy could be established by using an organic overlay phase to capture the hydrophobic target pigments. This would prevent capacity limits and possible negative effects on production cells caused by excessive membrane deposits. With suitable filtration devices, the overlay phase could be exclusively utilized and retained in the production bioreactor, significantly reducing the volume relevant for downstream processing.

Consequently, our research adds to the existing literature by providing an additional set of tools and strategies. The presented biological-and technical foundation may be effectively used and extended in future endeavors not only for VIO synthesis but also for the broader field of consortium-based biomanufacturing in general.

## Concluding remarks

Modular co-culture-based production platforms could be an important expansion of the existing toolbox for bioprocess development. To fully exploit the capabilities of these synthetic production communities, it is essential to effectively translate the designed division of labor within a suitable process engineering framework. The VIO production example may serve as a guiding idea illustrating the pros and cons of such technology. Here, we focused on leveraging an interconnected production co-culture by implementing individual temperature optima and tailored process designs. Other examples may deal with the installation of other individual cultivation optima that are essential to get the best out of the synthetic production consortium.

### Supplementary Information

Below is the link to the electronic supplementary material.

Supplementary file1 (PDF 896 kb)

## Declarations

### Data availability

The data generated and analyzed during this study are available from the corresponding author upon reasonable request.
