# Supplementary material for: Synthetic co-culture in an interconnected two-compartment bioreactor system: violacein production with recombinant E. coli strains
Source: Bioprocess Biosyst Eng. 2024 Apr 16;47(5):713–24. doi: 10.1007/s00449-024-03008-1 (PMC11093872; doi:10.1007/s00449-024-03008-1)
Supplement: Supplementary file 1 — Supplementary file1 (PDF 896 kb) [file 449_2024_3008_MOESM1_ESM.pdf]

Supplementary Information

Synthetic co-culture in an interconnected two-compartment bioreactor system: violacein production with recombinant *E.coli* strains

Tobias Müller[1] (ORCID: 0000-0002-9696-0254)

Simon Schick[2]

Jan-Simon Klemp[1]

Georg A. Sprenger[2] (ORCID: 0000-0002-7879-8978)

Ralf Takors[1] * (ORCID: 0000-0001-5837-6906)

[1] Institute of Biochemical Engineering, University of Stuttgart, Stuttgart, Germany

[2] Institute of Microbiology, University of Stuttgart, Stuttgart, Germany

* Corresponding author: Prof. Ralf Takors (ralf.takors@ibvt.uni-stuttgart.de). Institute of Biochemical Engineering. University of Stuttgart, Allmandring 31, 70569 Stuttgart, Germany.

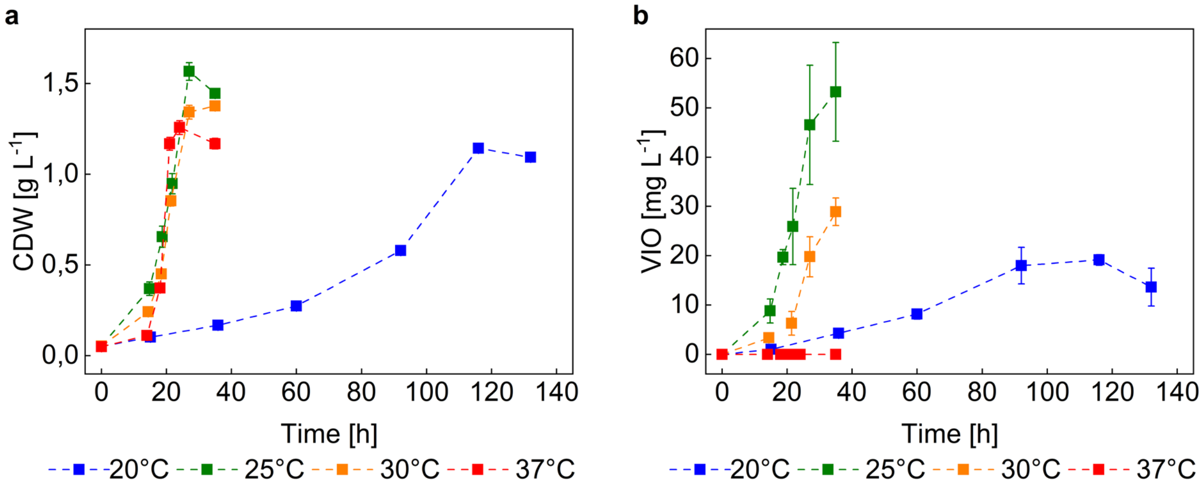

Fig. S1 Concentration profiles of **a** biomass (CDW) and **b** crude violacein (VIO) of induced TRP-4 strain in shaking flask cultivations (5 g L^{-1} arabinose and 0.1 g L^{-1} anthranilate) at different temperature settings. Error bars indicate the standard deviation of biological triplicates

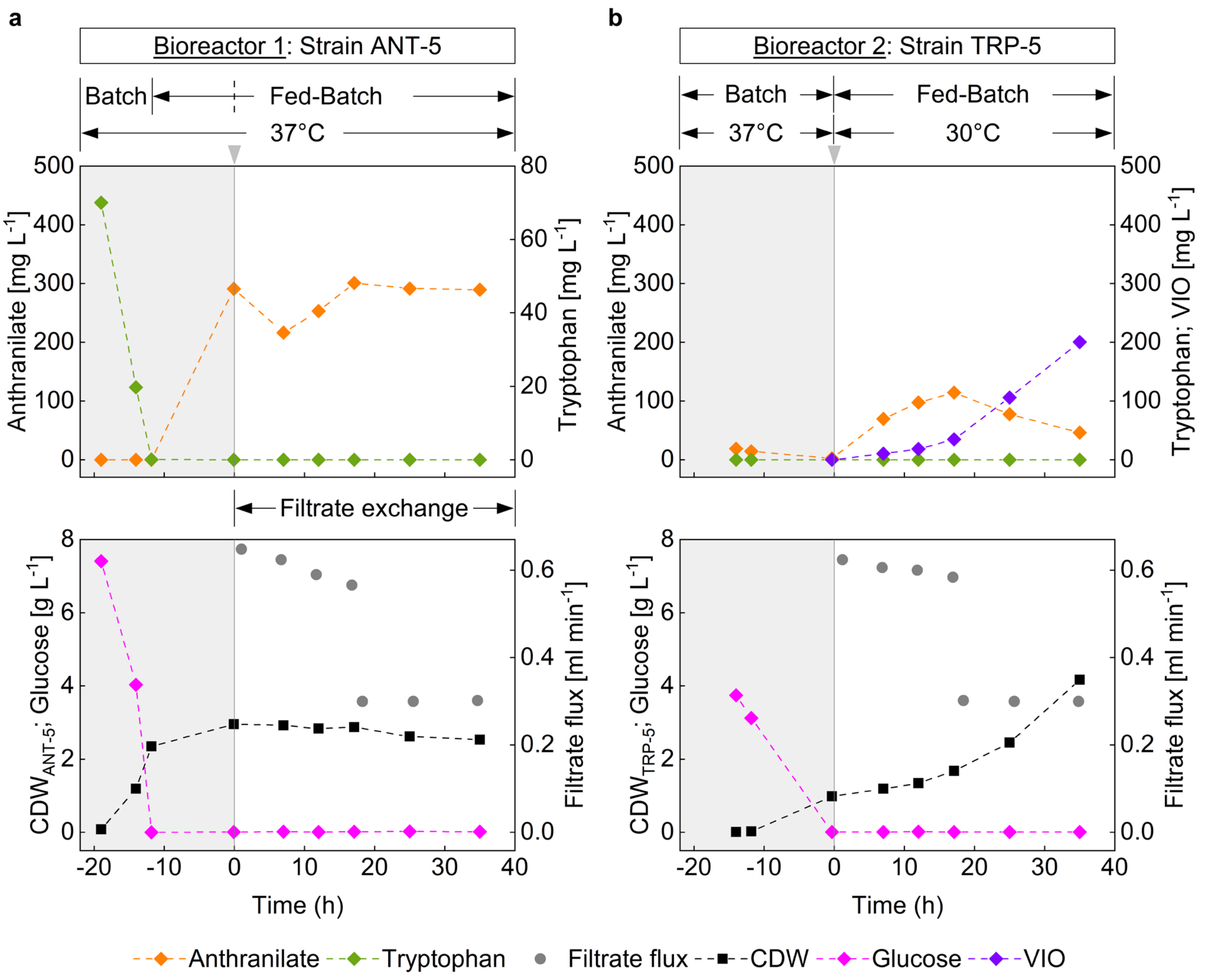

Fig. S2 Process Strategy III - Time courses of biomass (CDW)- and extracellular metabolite concentrations in fed-batch production of crude violacein (VIO) by a spatially separated co-culture in an interconnected two-compartment bioreactor system. **a** Representation of the concentration curves, as well as the filtrate flux from bioreactor 1 (Harboring strain ANT-5). The dashed line within the fed-batch phase indicates the time point of the D-glucose feed-rate adjustment. **b** Representation of the concentration curves, as well as the filtrate flux from bioreactor 2 (Harboring strain TRP-5). The gray areas correspond to the supplemented cultivation phase before filtrate exchange, inductor addition (grey arrows) and temperature change in bioreactor 2. For process details, see *Process Strategies II and III* in the *Materials and methods* section

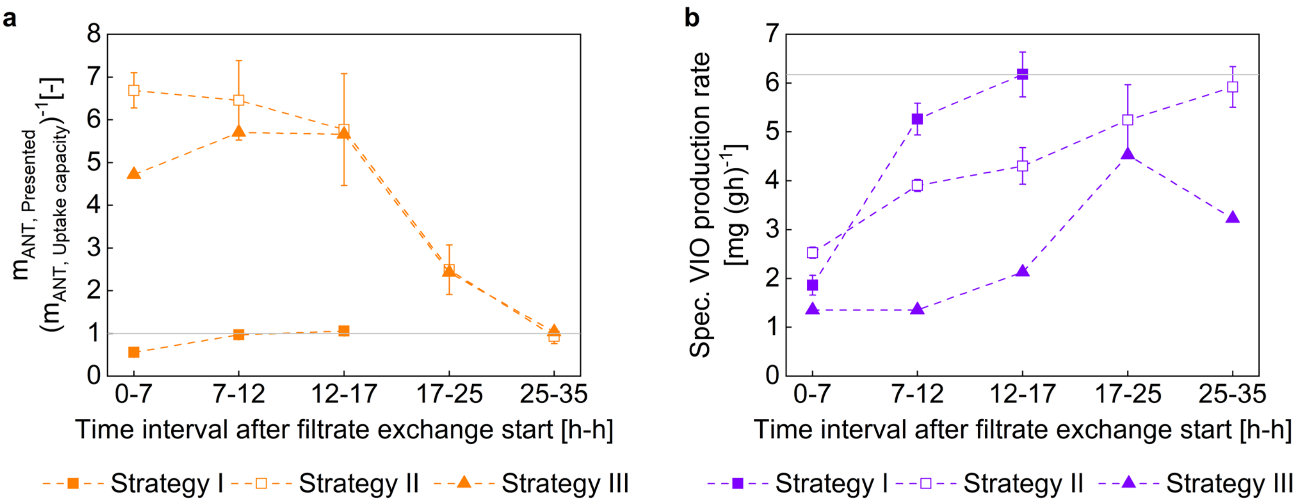

Fig. S3 Quantitative consideration of relative anthranilate (ANT) dynamics and absolute crude violacein (VIO) production rates of TRP-5 in bioreactor 2 after the initiation of the induced filtrate exchange period. **a** Calculated mass ratio of the presented ANT and the uptake capacity within specific time periods (≙ $f_{ANT,\ Ratio,\ R2}$). The horizontal reference line marks the balanced state of both elements. **b** Biomass-specific VIO production rates within specific time intervals. The horizontal reference line corresponds to the highest identified value. The filled squares represent the ratios and rates of Process Strategy I (See Fig. 3). The blank squares represent the ratios and rates of Process Strategy II (See Fig. 4). The filled triangles represent the ratios and rates of Process Strategy III (See Supplementary material, Fig. S2). Time intervals are given in full hours. Details on calculation can be found in the *Materials and methods* section. Error bars indicate the standard deviation of biological duplicates (Strategies I and II)

Extracellular tryptophan and anthranilate concentrations were quantified using an UHPLC system (UltiMate 3000-Series; Dionex; Thermo Fisher Scientific Ind., USA). The injection volume of the supernatant samples was 20 µL, the samples were stored in an autosampler at 5°C. The liquid chromatography method relied on a C-18 column (Luna C18[2], 5µm, 250×4.6 mm; Phenomenex Inc, USA) at 40°C, and the use of two mobile phases. Mobile phase A consisted of H_2O + 0.1 % trifluoroacetic acid (TFA), Mobile phase B consisted of methanol + 0.1 % TFA. A constant flowrate of 0.5 ml min^{-1} was applied. Starting with a mobile phase ratio of 98% Mobile phase A and 2% Mobile Phase B, the solvent gradient shifted as specified in Table S1. Using a Diode Array Detector, tryptophan was detected at a wavelength of 280 nm and anthranilate at a wavelength of 330 nm. Quantification was based on external standards.

Table S1 Time course of the mobile phase gradient for HPLC measurement of tryptophan and anthranilate

Method time [min]	Mobile phase B (Methanol+0.1%TFA) fraction [%]
1	2
2	20
12	70
23	90
25	98
27	98
27.5	2
30	2
